# Quantitative dynamics of VE-cadherin at endothelial cell junctions at a glance: basic requirements and current concepts

**DOI:** 10.15190/d.2016.10

**Published:** 2016-08-24

**Authors:** Jochen Seebach, Jiahui Cao, Hans Joachim Schnittler

**Affiliations:** Institute of Anatomy and Vascular Biology, Westfälische Wilhelms-Universität Münster, Münster Germany

**Keywords:** Quantifying endothelial junction dynamics

## Abstract

Intercellular junctions of the vascular endothelium are dynamic structures that display a high degree of plasticity, which is required to contribute to their regulation of many physiological and pathological processes including monolayer integrity, barrier function, wound healing and angiogenesis. Vascular endothelial cadherin (VE-cadherin) is connected via catenins to the actin cytoskeleton, both of which are key structures in endothelial junction regulation, and thus are the focus of much investigation. Fluorescence-based live cell imaging is the method of choice to study dynamic remodeling in living cells. Although these methods have been successfully applied to many cell types, investigations of endothelial junction dynamics were for a long time limited as they are largely resistant to transfection using many classical protocols. Application of virus-based gene transduction techniques, together with advanced microscopy, now allows both sufficient expression of fluorescence tagged junction-localized proteins in the endothelium and time-lapse recording over long periods. Using highly spatiotemporally resolved fluorescence microscopy it turned out that endothelial junctions display extensive junction heterogeneity at the subcellular level; a fact that largely limits automated quantification by available software. Recent work describes open software tools to quantitatively analyze large amounts of fluorescence-based image data in either single or confluent epithelial and endothelial cells. Based on quantitative VE-cadherin and actin dynamics novel key players, mechanisms and concepts have been suggested that control endothelial junction dynamics. Here we aim to summarize the recent developments in the field.

## SUMMARY

1. *Introduction*
2. *Methods to visualize endothelial cell junction dynamics *
3. *Endothelial junction dynamics analyzed by fluorescence techniques *
4. *Dynamics of VE-cadherin analyzed by spinning disc microscopy *
5. *Conclusion*

## 1. Introduction

The vascular endothelium is a highly plastic tissue that covers the inner surface of the entire cardiovascular system. It regulates many physiological functions, such as control of blood pressure and perfusion and balancing coagulation/anticoagulation processes. The endothelium is the critical boundary surface that separates the blood from the interstitial space of tissues, a feature that is generally referred to as endothelial barrier function. Cell integrity and barrier function depend on the formation of cell junctions in an organ and vascular segment specific manner^1,2^. A systemic loss of cell junction integrity contributes to the pathogenesis of life threatening diseases such as Ebola hemorrhagic fever^3^ and bacterial sepsis^4-7^, demonstrating a central role of cell junctions in both physiology and pathology. Endothelial cell junctions, in contrast to epithelial junctions, are complex in nature and display components of tight, adherens and gap junctions^8-11^. To accomplish the diverse organ and vascular segment specific demands of their multiple roles in physiology, development and diseases such as inflammation, wound healing, angiogenesis and tumor angiogenesis, cell junctions needs to dynamically respond on different time scales; ranging from minutes to many hours.

A main structural and regulatory molecule in endothelial junctions is vascular endothelial (VE)-cadherin that connects endothelial cells to each other in a calcium-dependent manner^12,13^. VE-cadherin is critical in controlling endothelial junction dynamics as it provides the junction backbone, and is expressed in all types of vascular endothelial cells^12,14^. The cytosolic domain of VE-cadherin directly interacts with beta-catenin, gamma-catenin and p120. While p120 prevents VE-cadherin endocytosis, β- and γ-catenin bind to α-catenin, which in turn mediates the interaction of the cadherin/catenin complex with the actin cytoskeleton^15-17^.

**Figure 1:**
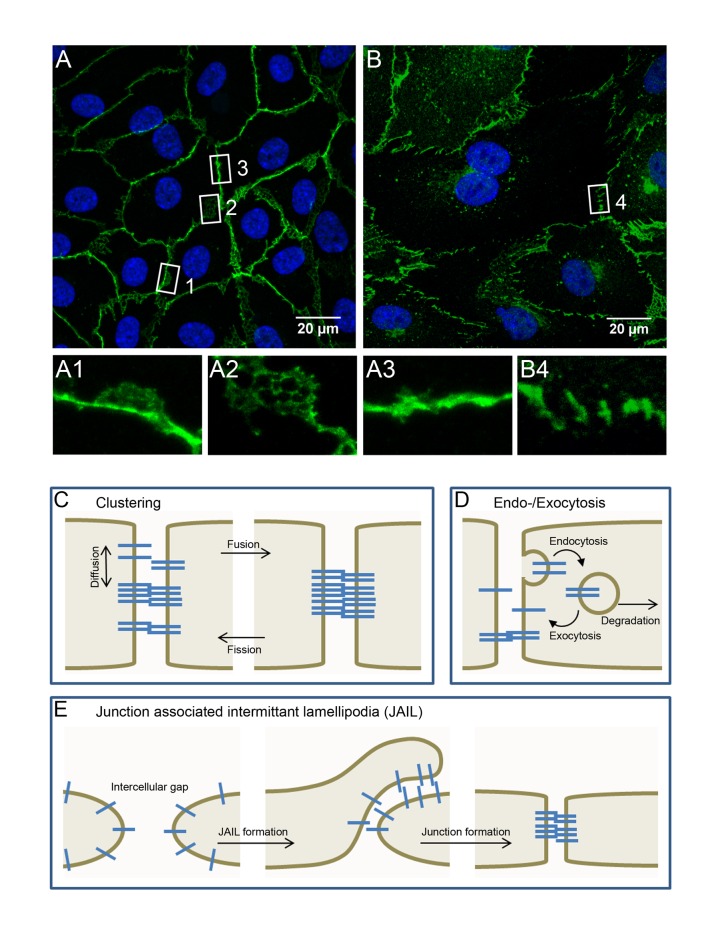
Heterogeneity of endothelial junctions in HUVEC cultures Immune fluorescence microscopy of confluent (A) and subconfluent (B) endothelial cells stained with anti VE-cadherin (green) and DAPI (blue). Cropped areas show the diverse super-structural organization of endothelial junctions. This includes plaque-like structures (A1), reticular networks (A2), linear junctions (A3) and interrupted patterns (B4). These diverse superstructures are constitutively reorganized by dynamic VE-cadherin remodeling via processes such as fusion and fission of VE-cadherin clusters (C), endo- and exocytosis (D) and by locally appearing junction associated intermittent lamellipodia (JAIL), which develop at junction sites with a low VE-cadherin concentration (E).

It is generally proposed that control of cell adhesion, barrier function, cell migration and angiogenesis critically depend on the direct and indirect interaction of VE-cadherin with actin filaments, as both structures are remodeled under certain stimulations^18-22^. When examined by classical laser scanning microscopy (LSM) VE-cadherin displays diverse staining patterns, with linear, reticular, interrupted and plaque morphologies being evident (Figure 1 [A, B]). Accordingly, junction dynamics in the endothelium is obviously not uniform, but rather it display a wide heterogeneity even in one cell within a monolayer^23^. Super resolution microscopy of epithelial and endothelial adherens junctions indicates the existence of individual cadherin clusters^20,24-26^ whose size seems to be dynamically regulated by fusion and fission^20,25^, (**Figure 1** [C]).

The molecular mechanistic basis for subcellular junction regulation remains largely unknown, despite the many mechanisms related to cell signaling and junction remodeling, including VE-cadherin and the diverse cytoskeletal filament system, that have been discussed in certain reviews^16,17,27-30^. While biochemical or biophysical measurements, such as impedance spectroscopy, give mean values of dynamic barrier function regulation, high time resolution live cell imaging additionally uncovers information regarding spatiotemporal regulation at the subcellular level. In addition, the temporal sequence of short duration events are easily perceived in time lapse movies, while those same phenomena are hard to distinguish in snap shots. One such structure are junction associated intermittent lammellipodia (JAIL, see sections below for additional details), which are actin-driven and cause the formation of new VE-cadherin adhesion. In confluent cultures, in which JAIL are small and short lasting, these structures can only be clearly identified in time lapse recordings (available at http://link.springer.com/article/10.1007/s00418-015-1357-8). Therefore, live cell imaging of endothelial cell junction dynamics, followed by quantitative analysis, can significantly upgrade the information output of experiments, but requires methodologically sound techniques and specialized equipment.

## 2. Methods to visualize endothelial cell junction dynamics

### Basic problems and requirements for analysis of endothelial junction dynamics

The method of choice to study spatiotemporal dynamics in cells and cell junctions at the subcellular level is live cell imaging of fluorescence tagged proteins. Excellent work conducted during the last years describes the available techniques and methods^31,32^. This includes the generation and expression of fluorescence tagged proteins^33,34^, and the development of software tools^35-37^. In addition, advanced microscopic techniques, such as spinning disc and confocal laser microscopy, Förster or fluorescence resonance energy transfer (FRET), fluorescence recovery after photobleaching (FRAP), single molecule tracking (SMT) and structured illumination microscopy (SIM) have been successfully applied to answer many scientific questions in many different cell types. However, expression of fluorescence tagged proteins in the endothelium is not a simple approach as most endothelial cells, and in particular primary isolates such as HUVEC, resist transfection using classical methods^38^. Also, electroporation of suspended cells bathed in a plasmid containing medium has been reported to cause cell impairments^39^ that in turn can perturb cell junction maturation and thus these cells might not exhibit characteristic endothelial cell adhesion and barrier features.

### Virus-based gene transfer is sufficient to express fluorescence tagged proteins in the endothelium**

**Figure 2:**
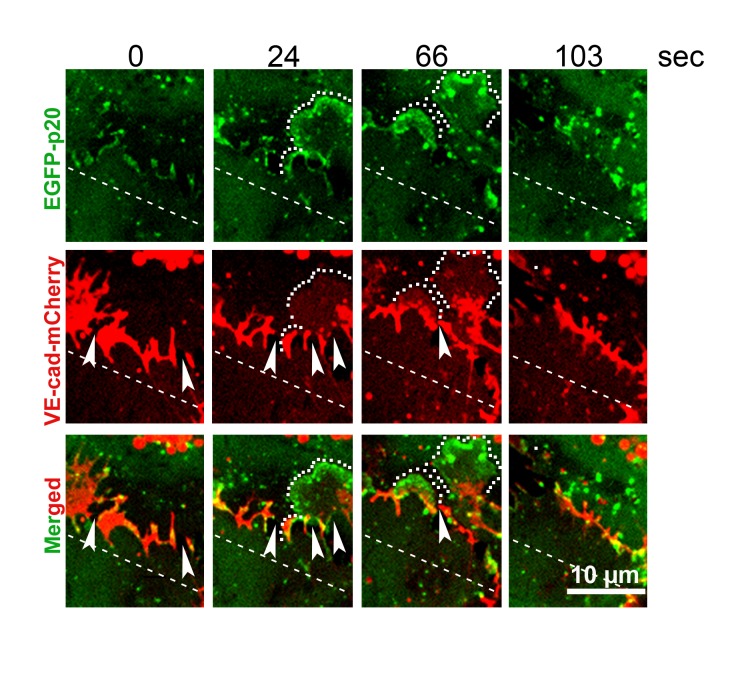
VE-cadherin dynamics is driven by ARP2/3 complex controlled JAIL formation. Time-lapse recording of sub confluent HUVECs that co-express both VE-cadherin-mCherry and EGFP-p20, a component of the ARP2/3 complex. VE-cadherin remodeling is initiated by ARP2/3-controlled and actin-driven formation of JAIL (dotted line), which then overlap adjacent membranes allowing trans-dimer engagement of free floating VE-cadherin in the membrane (VE-cadherin plaques), followed by JAIL retraction that subsequently clusters and incorporates VE-cadherin into the junctions (reproduced from our previous work^41^ (Abu Taha A et al. Mol Biol Cell 2014, 25: 245-256) with permission).

A suitable and meanwhile frequently applied alternative for gene expression in endothelial cell lines as well as primary cell cultures is the use of replication-deficient recombinant lenti-, retro- or adeno-viruses^40^. Since many lentivirus-based vectors include an integrase, the generation of constitutively expressing cultures is also relatively easy to perform. Although the generation and production of replication-deficient recombinant viruses is time and cost intensive it gives excellent transduction efficacy and expression levels can be controlled as well. A few examples in which, viral-based gene transduction was efficiently applied include studies where up to 90% gene transduction was demonstrated for fluorescence tagged caveolin1, proteins of the ARP2/3 complex, LifeAct, GTPases, VE-cadherin, β-actin, β-catenin and γ-catenin^41,42^. Furthermore, carefully performed virus titration is useful in controlling the protein expression level in the endothelium to achieve either moderate or even strongly overexpressed protein levels. After infection, gene expression from lentiviruses is typically sufficient after three to four days and is best suited to performing spinning disc microscopy over a period of hours^23,41,43^. Adenoviral mediated gene expression is usually faster, reaching high expression levels after approximately 10 to 16 hours post infection, as demonstrated for the Rho-GTPases and VE-cadherin-EGFP^22,23,44,45^. Together, virus-based gene transduction is currently the most successful approach available and the method of choice to obtain reliable, reproducible and controlled protein expression levels in the endothelium. An example showing expression of VE-cadherin-mCherry and EGFP-p20 are shown in**Figure 2**. However, any type of exogenous protein expression in cultured cells has the potential to exhibit toxic effects. Therefore, when using virus mediated gene transfer methods, the respective controls need to be carefully planned. Further, to test if the expression of a particular fluorescence tagged protein disturbs normal cell behavior, functional tests are highly recommended. For example, moderate expression of VE-cadherin-EGFP in the endothelium did not cause cells to behave differently compared to native untreated endothelial cells when tested in a scratch assay. This also holds true for testing the barrier function of VE-cadherin-EGFP or caveolin-1-EGFP expressing cells upon stimulation compared to native non-transduced cells^41,46^. Those functional tests verify the regular behavior of cells even during fluorescence tagged protein expression. In other cases expression can be used to study the impact of protein concentration on cell functions, as demonstrated by overexpression of VE-cadherin, which leads to clearly reduced cell motility^41^. Therefore, we suggest choosing functional tests for ensuring regular activity of cells expressing fusion proteins, rather than simple expression of an EGFP or mCherry molecule by itself, because those proteins might also cause artifacts.

### Time lapse recordings of fluorescence tagged proteins at endothelial cell junctions

Fluorescence-based live cell imaging is a powerful approach to study protein dynamics and to a certain degree also protein interactions due to its high sensitivity and spatiotemporal resolution. Time-lapse recordings of fluorescence microscopy, however, is limited by photobleaching and phototoxicity due to extensive light excitation. Photobleaching results in reduction or even total loss of the fluorescence signal, while phototoxicity relates to the generation of reactive oxygen species (ROS) that in turn can alter protein- , lipid- and nucleic acid-function^47^. Investigated cells might, therefore, become impaired and exhibit intercellular disintegration, artificial protein remodeling and/or inactivation of junction molecule dynamics. These problems might then generate misleading findings and lead to incorrect interpretations, and thus have to be taken in consideration. Fortunately, most of these drawbacks can be limited by carefully determining the a) minimal required illumination time to avoid production of high amounts of oxygen radicals, b) minimal image acquisition frequency, which gives the cells the chance to degrade oxygen radicals when combined with anti-oxidant treatment, c) usage of high sensitivity photo-detectors (e.g GaAsP detectors) for confocal laser microscopy, which allows less excitation power to be used, and/or d) use of other advanced microscopy techniques, such as spinning disc microscopy (SPDM)^48^. Use of these techniques, together with careful adjustment and optimizations of the settings, is in our experience the best approach for performing live cell imaging in the endothelium over many hours without pronounced toxicity or significant loss of signals.

## 3. Endothelial junction dynamics analyzed by fluorescence techniques

There are a number of excellent techniques that can be used to study cell junction dynamics, including protein turnover, protein displacement or the formation of subcellular structures such as JAIL. Established methods include single molecule tracking, fluorescence correlation spectroscopy (FCS), FRAP, classical confocal laser microscopy, spinning disc microscopy and, with increasing success, high resolution SIM. These techniques have been successfully applied to the study of cytoskeletal dynamics (e.g. actin and vimentin), intercellular adhesion and cell-matrix interaction in various cell types^49-53^.

### Single molecule (particle) tracking

Single Molecule Tracking (SMT), also referred as Single Particle Tracking (SPT) or Single Dye Tracking (SDT), uses stably tagged molecules (e.g. organic dyes, fluorescent proteins, quantum dots, gold particles) that can be directly followed in real time using high resolution fluorescence microscopy (Nanoscopy). SMT is valuable for analyzing heterogeneities and molecule dynamics, which are usually obscured in averaged data sets^54^, at the nanoscopic level. For further information we refer the interested reader to several excellent manuscripts that have been recently published^54-57^.

SMT techniques have been successfully applied to study the mobility of free-floating E-cadherin^58,59^ and VE cadherin^60^ in the plasma membrane of living cells. The authors concluded that the lateral motility of cadherins is limited by the membrane cytoskeleton either by binding to the cytosolic domain cadherin (tethering) or by acting as a fence for unbound cadherin (corralling). In addition, it was shown, that E-cadherin forms small oligomers/clusters before the proteins became incorporated into the cell junctions^58^. So far these techniques have already revealed many details about the complex dynamcis of cadherins outside of the cell-cell contact regions. It is likely that future SMT studies examining cadherins incorporated into the cell junctions will give further valuable information about regulatory mechanisms.

### Fluorescence recovery after photobleaching**

FRAP is a suitable method for studying dynamic remodeling of fluorescence tagged molecules at the subcellular level, including at cell junctions. A region of interest is bleached using a high laser power without destruction of the molecule itself (bleaching), followed by time-dependent image acquisitions to measure the reappearance of fluorescence in the bleached area (recovery). Based on the biological question FRAP can be successfully used to estimate parameters such as lateral diffusion, protein exchange (e.g. by endo and exocytosis) or cytoskeletal remodeling. For example, actin recruitment to cell junctions due to increases in cAMP resulted in reduced VE-cadherin mobility^61^, which is accompanied by stabilization of endothelial barrier function^62,63^. Furthermore, a fluorescence tagged fusion protein consisting of VE-cadherin and α-catenin (VE-cad-a-C-GFP) expressed in endothelial cells displayed a reduced mobility in a latrunculin-dependent manner^64^. In addition, nectin, another adherens junction protein that forms trans-dimers between adjacent cells, interacts via afadin and drebrin with F-actin. FRAP analyses show a higher mobility of afadin in drebrin depleted cells^65^. Together, these FRAP data indicate that interaction with the actin cytoskeleton significantly modulates VE-cadherin dynamics.

It is well known that surface expression of junction proteins is dynamic, and like other membrane proteins they undergo both endo- and exocytosis (**Figure 1** [D]). Endocytosis of VE-cadherin was described after treatment of cultured cells with bradykinin or even during angiogenesis *in vivo* in a phosphotyrosine - 658 and - 685 dependent manner^66-68^. FRAP analyses demonstrated an impaired recovery of VE-cadherin 1) in the presence of endocytosis inhibition, 2) after expression of tyrosine 658 mutated VE-cadherin and 3) after activation of Notch signaling^66,67^. In contrast, when 3 amino acids that were shown to be necessary for endocytosis are mutated in the juxtamembrane cytoplasmic domain of VE-cadherin, there is no effect on the recovery time following similar treatments^69^.

FRAP parameters have also been correlated with different VE-cadherin distribution patterns and endothelial barrier function. For example expression of epithelial cadherin (E-cadherin) in endothelial cells shows a heterogeneous distribution with low- and high-density segments. The low density adherens junctions displayed a spotty cadherin density, with higher cadherin mobility and reduced barrier function. In contrast, stimulation of endothelial cells with plasminogen activator inhibitor-1 (PAI-1)^70^ or thrombin^23^ reduces the endothelial barrier function, but the mobile fraction remained unchanged (after PAI treatment)^70^ or were even transiently reduced (after thrombin application)^23^. In addition, certain changes in VE-cadherin dynamics are necessary for the opening and recovery of endothelial junctions after thrombin treatment^23,71^. Together the data demonstrate that FRAP is a highly effective method to study local subcellular protein dynamics at cell junctions. Although FRAP gives valuable information about dynamics, it is limited to particular sites of endothelial junctions and estimations of heterogeneity are hard to perform (**Figure 1** [E]). This is particularly the case in subconfluent cells in which the dynamics of VE-cadherin and actin are high, due to frequent and large JAIL formation^72^. In other words, when choosing FRAP analyzes to estimate dynamics of junction proteins in the endothelium, junction heterogeneity must be taken into consideration and the regions of interest must be carefully selected in such a way as to be statistically relevant. Such heterogeneity must also be taken into consideration when analyzing junction dynamics in the endothelium using photo-activatable molecules. For example, in a recent paper rac-1 was suggested to stabilize trans-dimer formation of VE-cadherin in mature endothelial cell junctions by counteracting acto-myosin mediated contraction^73^. However, in this study cell junction heterogeneity was not taken in consideration, and thus the entire impact of this study is still unclear. In summary, the data demonstrate that a simple correlation between junction protein mobility and endothelial barrier function and VE-cadherin dynamics does not exist.

### 4. Dynamics of VE-cadherin analyzed by spinning disc microscopy

Dynamic remodeling of VE-cadherin and catenins (i.e. VE-cadherin/catenin complexes) and their interaction with actin filaments are critical in controlling cell junction integrity, permeability and migration activity. Interaction between the VE-cadherin/catenin complex and actin filaments includes many regulatory mechanisms important in physiology and pathology, including activation of kinases, phosphatases, Rho-GTPases and phospho-caveolin-1, to name a few^16,22,46,74,75^. Published data and the associated mechanism identified to date have mostly been obtained from time-dependent snap shots^76^. Recent papers, however, have for the first time examined VE-cadherin dynamics directly in the endothelium using live cell imaging of fluorescence tagged VE-cadherin^23,41,77^. These papers demonstrate a quick and constitutive dynamic remodeling of VE-cadherin that was also strongly related to cell density. In confluent cultures, which comprise small polygonal cells with a small cell junction perimeter and less overall cell motility, VE-cadherin largely appears as a linear densely packed pattern, but also displays reticular, plaques and small interruptions with less dynamic remodeling. This VE-cadherin pattern can be compared to that of venous endothelium *in situ*. In contrast, VE-cadherin dynamics and cell migration activity is much higher in growing cells and the VE-cadherin pattern quickly switches between linear, interrupted, reticular and plaque like structures (**Figure 1** [A, B], movie)^23,41^. The molecular background for the dramatically different VE-cadherin dynamics in confluent versus growing cultures could at a very fundamental level be directly related to the cell border length. This is due to the equal and cell growth-independent amount of VE-cadherin present in confluent and subconfluent (growing) endothelial cells^41,78^. Given that a constant amount of VE-cadherin is distributed at the cell junctions, the long cell junction perimeter in growing cells thus contains a reduced relative VE-cadherin concentration at junction sites, while the same amount of VE-cadherin is more concentrated in the junctions of small polygonal confluent cells with a reduced perimeter. Intriguingly, growing cells with high VE-cadherin dynamics display high cell motility, while confluent cells with reduced VE-cadherin dynamics are much less motile. This phenomenon could directly be related to the development of JAIL, which are actin-driven and ARP2/3 complex-dependent actin networks that forming plasma membrane protrusions appearing at subcellular junction sites were VE-cadherin is absent or displays a reduced relative concentration^41^. JAIL form new VE-cadherin adhesion sites and thus drive VE-cadherin dynamics. The detailed mechanism occurs in a sequential order and was analyzed by co-expression of both VE-cadherin-mCherry and EGFP-p20, a component of the actin related protein complex 2/3 (ARP2/3), which controls the actin network turnover of plasma membrane protrusions, including lamellipodia^79^ and JAIL^41^. In particular, at sites with a lower VE-cadherin concentration small locally restricted JAIL are formed that overlap adjacent cells, and in turn induce engagement of free floating VE-cadherin in the membrane, and thus the formation of VE-cadherin adhesion plaques^23,41^ (**Figure 1**, [A1, A2]). During JAIL retraction VE-cadherin clusters and becomes incorporated into the plasma membrane^41^. Thus, this process drives the high levels of VE-cadherin dynamics seen in growing cell cultures, where VE-cadherin interruptions are much more frequent than in confluent cells. The critical impact of VE-cadherin in overall cell junction dynamics was demonstrated by overexpression of VE-cadherin in growing (large perimeter) cells and by application of ARP2/3 inhibitors. Both manipulations blocked JAIL formation and decreased cell motility. It should be noted that JAIL differ significantly from classical lamellipodia, which regulate cell migration on a substrate requiring integrin mediated cell adhesion^80,81^. In contrast, JAIL form new VE-cadherin adhesion sites and control junction integrity and cell motility within a junction-forming cell layer. These mechanism could be uncovered using double expression of fluorescence tagged molecules and analysis by fluorescence live cell imaging, a procedure that allowed analyses of both spatial and temporal protein dynamics at the same time.

### Quantitative VE-cadherin and actin dynamics at endothelial cell junctions

The recent advancements in viral-based expression of fluorescence tagged molecules in primary isolates of the endothelium and advanced microscopic techniques such as spinning disc microscopy as well as the development of GaAsP detectors now allow generation of image sequences over relatively long time periods with thousands of images being generated. While time-dependent snap shots can be quantitatively analyzed by manual selection and the use of image analysis software, such as ImageJ, the analysis of long time-lapse recordings is impossible to perform manually. For such experiments quantitative analysis requires software tools that are capable of automatically selecting the regions of interest in image sequences, followed by further mathematical processing to extract the parameters of interest.

### Segmentation as a critical step in image analysis**

**Figure 3:**
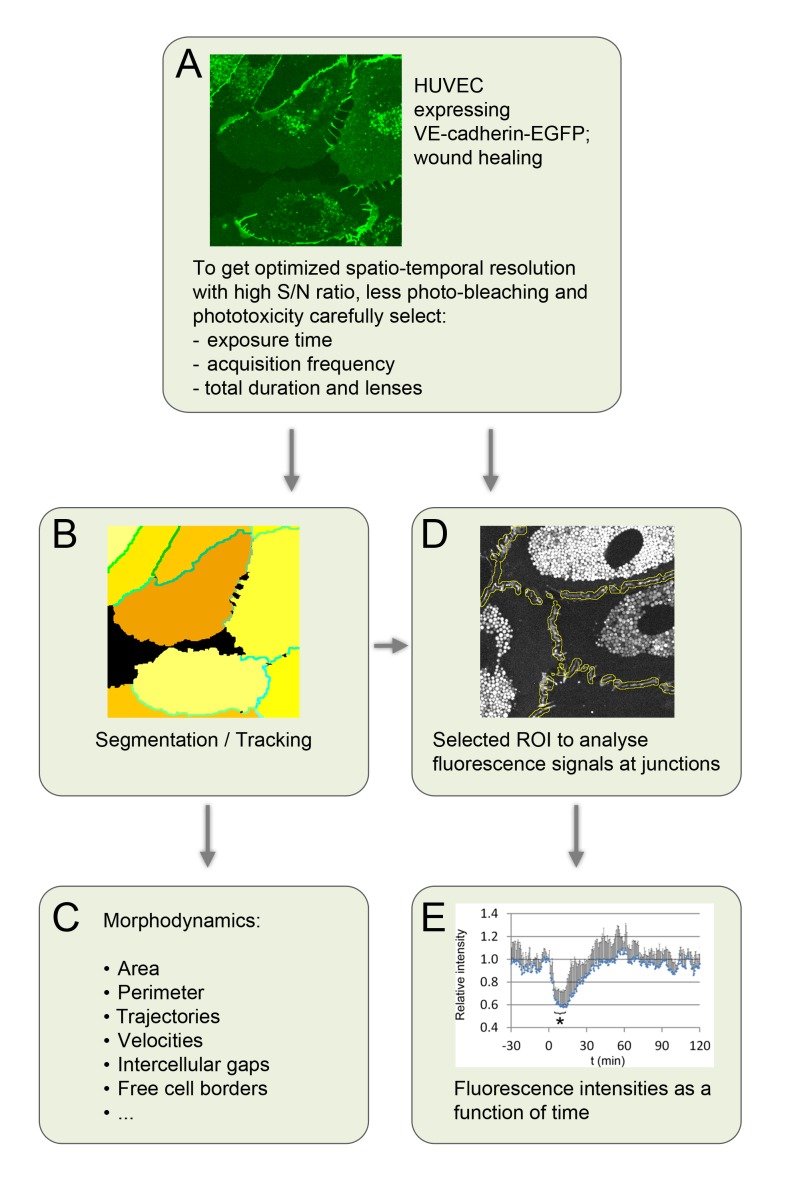
Scheme illustrating the work-flow for quantitative analyis of endothelial junctions and free cell border dynamics from image segmentations Image taken of HUVEC cultures that express VE-cadherin-EGFP after optimization of microscopic settings (A). The cell border extractor was applied and segments both cell junctions and individual cells, and subsequently assigns each cell and each cell junction a unique color-coded identification number (ID), (B). Many parameters can be extracted from the segmentations (C). The identified position of the color-coded intercellular junctions (B, different green colored lines) can be used to generate regions of interest (ROI) (D, yellow outlines) that in turn provide input parameters to quantify various parameters, for example fluorescence intensities as demonstrated for the EGFP-p20 intensity at endothelial junctions upon thrombin applications (E) (reproduced from our previous work^23^ (Seebach et al, Histochemistry and Cell Biology 2015, 144: 517-532) with permission).

Image segmentation and object tracking is a common approach to the analysis of dynamic processes in time-lapse sequences (**Figure 3** [A, B]). This approach has been widely used to analyze the morphological parameters of single cells, such as cell perimeter, cell area, cell motility or plasma protrusions, including lamellipodia and filopodia^82-84^ (**Figure 3** [C]). While the segmentation of fluorescence tagged structures in single cells can often be accomplished using a simple threshold analysis, the segmentation of confluent monolayers is still a challenging task. However, in recent years several excellent segmentation tools have been developed and implemented to analyze time lapse sequences of fluorescence tagged E-cadherin in cultured cell and in drosophila in order to study morphogenesis^85-87^. Those tools are excellently suited to quantify cell morphology and cell fate, but reveal few or no details regarding subcellular junction organization. An easy to use tool for quantifying subcellular protrusions in single cells, known as Automated Detection and Analysis of Protrusions (ADAPT), was recently developed and is available as open source software as well^82^.

Further, a novel image analyses tool especially developed to study quantitative endothelial cell junction dynamics called CellBorderTracker (CBT) has recently been developed. CBT consists of three basic tools: 1) CellBorderExtractor (CBE), which segments endothelial cells, 2) CellMapper (CM), which identifies and labels individual cells and each cell junction and 3) junction-kymograph (j-kymograph), which allows spatiotemporal analysis of entire cell junctions^23^ with the possibility to also identify junction heterogeneities^23^. The basic data obtained are well suited for the extraction of many secondary parameters, such as quantitative protein distribution, the generation of regions of interest, junction displacements, cell shape changes, migration velocities and the visualization of junction dynamics at the subcellular level, to name a few. In particular CBT is able to estimate subcellular activities, such as lamellipodia or JAIL formation, and determine the presence of intercellular gaps. The segmentation data provide a source for defining junctional regions of interest (jROI) (**Figure 3** [D]), which together with local fluorescence intensity can be used to determine the amount and dynamics of fluorescence tagged proteins even at the subcellular level^23,82^. Alternatively, dynamic remodeling can also be quantified by analyzing the differences in gray values for each pixel in a defined jROI^23,82^.

An example of the application of CBT to the quantitative analyses of spatiotemporal dynamics (i.e. time lapse sequences) of fluorescence tagged proteins at endothelial junctions involves remodeling of the VE-cadherin/catenin complex and actin in response to stimuli such as TNF-a, thrombin and histamine^18,19,21,88^. Although, the VE-cadherin remodeling is a dynamic process it has until recently been investigated mostly using snap shots in a time-dependent manner. Recent work by independent groups has demonstrated the dynamic remodeling of VE-cadherin and actin using live cell imaging to generate high resolution time lapse recordings^23,77^. These experiments demonstrated that thrombin application blocks JAIL formation by rebalancing rac-1/rho activity, which in turn leads to VE-cadherin remodeling, stress fiber and gap-formation with a resulting breakdown in barrier function. Resealing of the monolayer was induced by the reappearance of JAIL formations that in turn form new VE-cadherin adhesion sites and closed the thrombin-induced gaps.

### Subcellular heterogeneities of endothelial cell junctions**

Analysis of global parameters do not account for the local subcellular heterogeneities of cell junctions in endothelium. We strongly believe, and there is increasing evidence to support, that spatiotemporal diversity of cell junction regulations plays a more prominent role than generally expected. This includes roles in planar polarization, inter-individual cell communication within the cell monolayer and transmigration of tumor cells or leukocytes trough the endothelial cell layer. Further, subcellular heterogeneities at endothelial cell junctions exert a critical influence on characteristic changes associated with stimulation or cell growth^23,41^. This includes the formation of linear junctions, interrupted junctions, reticular networks and very transient VE-cadherin plaques^23^. These diverse structures then display diverse dynamics and differentially respond to stimulations, a fact that makes quantitative analysis of cell junction dynamics a challenging task.

**Figure 4:**
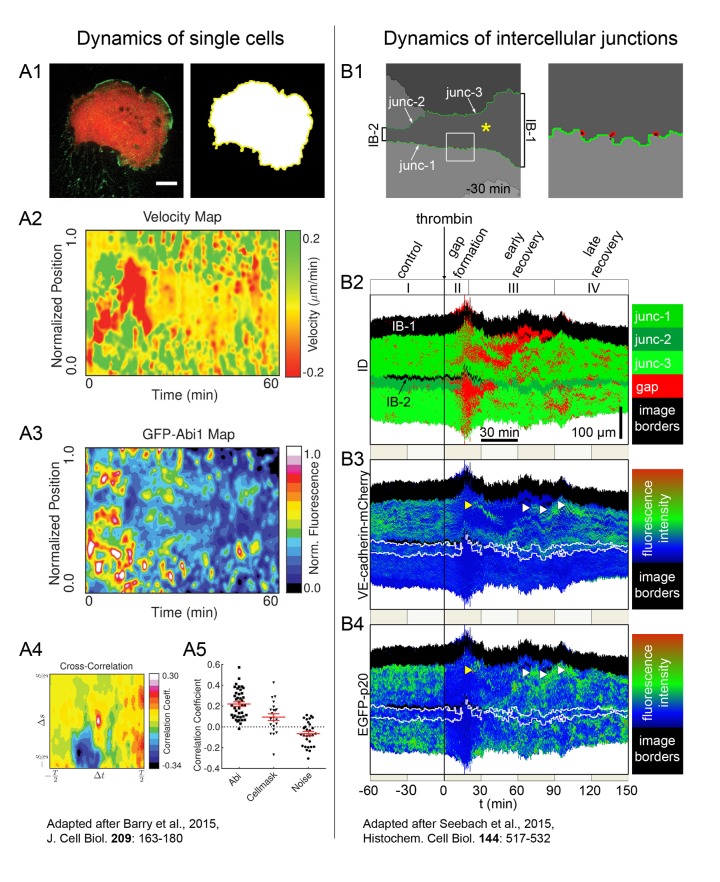
Spatiotemporal quantitative analysis of cell protrusions in single HT1080 cells and junction dynamics in confluent endothelial monolayers Subcellular protrusion heterogeneities at the plasma membranes of individual GFP-Abi1 expressing HT1080 cells (A) and at cell junctions of HUVEC expressing EGFP-p20 and VE-cadherin-mCherry (B) quantified by generation of kymographs that automatically follow the dynamic displacements of the cell borders or cell junctions, respectively. Fluorescence of GFP-Abi1 expressing HT1080 cells and their segmentation (A1). The depicted example is reproduced from^82^ (Barry DJ et al., J Cell Biol 2015; 209: 163-180) with permission, and shows kymographs of the membrane protrusion velocity (A2) and the GFP-Abi1 intensity (A3) in single HT1080 cells. A cross correlation of these maps (A4) allows testing of the statistical significance (A5) between these two parameters. The next example is reproduced from our previous work^23^ (Seebach et al, Histochemistry and Cell Biology 2015, 144: 517-532) with permission, and displays the quantitative dynamics of three endothelial cell junctions of one cell within a monolayer. Cell junctions were segmented using the CBE (B1) and intercellular gaps are identified as red spots, as seen in the cropped area (B1, right panel). j-kymographs display the distribution over time of intercellular gaps (B2, red), VE-cadherin-mCherry intensity (B3) and EGFP-p20 (B4) intensity at the indicated cell junctions upon thrombin application. The spatiotemporal opening of cell junctions and the subsequent recovery is clearly seen.

To document some of the spatial aspects of junction heterogeneity, signal intensities along boundaries of the segments can be plotted as a function of time using j-kymograph, in a manner comparable to a conventional kymograph^23,82^. In single migrating cells those maps have been useful to document subcellular protrusion activities at the cell borders of single cells (**Figure 4** [A])^82^. An example demonstrating a velocity map and a GFP-Abi1 appearance map is shown (**Figure 4** [A2, A3]). Furthermore, using a cross-correlation function that includes the velocity of membrane protrusions and the intensity of GFP-Abi1 gives clear information about local heterogeneities (**Figure 4** [A4, A5])^82^. In junction-forming confluent endothelial cell cultures we used comparable approaches (jKymographs) to visualize the spatiotemporal correlation between the presence of the EGFP-p20 (a component of the ARP2/3 complex) and VE-cadherin-mCherry dynamics upon stimulation with the serine protease thrombin, an agent that cause a transient decrease in endothelial barrier function. It was demonstrated that thrombin transiently inhibits ARP2/3 complex mediated JAIL formation^23,77^, followed by VE-cadherin-mCherry remodeling. The spatiotemporal dynamics can be visualized by jKymographs with separate spatiotemporal appearance of intercellular gaps (**Figure 4** [B1, B2]), VE-cadherin-mCherry gaps (**Figure 4**[B1, B2]), VE-cadherin-mCherry (**Figure 4**[B3]) and EGFP-p20 (**Figure 4**[B4])^23^. Together, the studies clearly demonstrate that subcellular heterogeneities in plasma membrane protrusions, such as filopodia, lamellipodia and JAIL, can be quantitatively estimated. Application of these tools will hopefully help us to better understand membrane dynamics and its regulation in more detail in the future.

## 5. Conclusion

In the last decade scientists have developed a number of ideas and models to understand endothelial cell junction dynamics, and particularly the dynamics of VE-cadherin, and their role in controlling cell integrity under physiological and pathological conditions. However, the regulation of many dynamic processes that control endothelial integrity, barrier function and shape changes are still unexplained. Viral expression systems now allow sufficient expression of fluorescence tagged proteins in the endothelium, and subsequently the performance of fluorescence live cell imaging to investigate and understand junction dynamics. The diverse fluorescence-based methods like FRAP, FRET, SMT, TIRF, SPDM and others that have been established now provide sufficient tools to study the molecular details of junction dynamics in the endothelium. However, understanding spatiotemporal diversity remains a challenging task as locally applied techniques, such as FRAP and FRET, deliver only locally restricted information, and cannot be simply transposed to the other parts of the cells. This becomes even more complicated as those local phenomena most probably depend upon local signaling and regulation. One possibility to better understand both local regulation and local structural remodeling involves tracking of entire cell junctions followed by subcellular analyses. Two nice open source software packages, ADAPT for quantitative analysis of subcellular heterogeneities in single cells^82^ and CBT for analyses of cell junctions in confluent endothelial cells, are now available^23^. Application of both tools will help to understand junction dynamics in more detail in the future. Furthermore, combined investigations using quantitative spatiotemporal dissolved high resolution fluorescence microscopy (e.g. two color approaches) together with biophysical methods, such as impedance spectroscopy, will allow us to not only directly observe the sequential steps of junction protein dynamics but also allow causal mechanistic conclusions to be drawn.

## Bullet Points

**◊ Fluorescence-based live cell imaging is the method of choice to study dynamic remodeling in living cells**

**◊ Live cell imaging uncovers the mechanisms of VE-cadherin dynamics not visible within snapshots**

**◊ Endothelial live cell imaging can be performed by expression of fluorescent tagged proteins, by using lenti-viral and/or adenoviral gene transfer**

**◊ Advanced microscopic techniques open new experimentations**

**◊ Novel software tools are available to quantify endothelial cell junction dynamics and detailed dynamcis of single cells**
